# Effect of dietary fiber and tributyrin on nursery pig health, performance, bacteriome, and volatile fatty acid concentration

**DOI:** 10.1093/jas/skag302

**Published:** 2026-09-21

**Authors:** Elizabeth M Due, Olivier Munezero, Eric R Burrough, Thomas E Burkey, Samodha C Fernando, Nicholas K Gabler

**Affiliations:** Department of Animal Science, Iowa State University, Ames, IA 50011, United States; Department of Animal Science, University of Nebraska-Lincoln, Lincoln, NE 68583, United States; Department of Veterinary Diagnostic and Production Animal Medicine, Iowa State University, Ames, IA 50011, United States; Department of Animal Science, University of Nebraska-Lincoln, Lincoln, NE 68583, United States; Department of Animal Science, University of Nebraska-Lincoln, Lincoln, NE 68583, United States; Department of Animal Science, Iowa State University, Ames, IA 50011, United States

**Keywords:** microbiome, volatile fatty acids, intestinal health, ETEC

## Abstract

Dietary fiber and volatile fatty acid (VFA) metabolites have been shown to improve intestinal health and reduce post-weaning diarrhea in pigs. Therefore, the current study was conducted to evaluate the impact of insoluble and soluble dietary fiber sources and butyrate on the intestinal health and growth performance of nursery pigs. It was hypothesized that insoluble and soluble dietary fiber sources or butyrate supplementation would improve nursery pig performance and enteric health. Over two replicates, a total of 610 weaned pigs were assigned to one of five dietary treatments (*n* = 17 pens/treatment): 1) control diet (CON), 2) wheat bran (WB), 3) beet pulp (BP), 4) corn distiller’s dried grains with solubles (DDGS), and 5) CON plus 0.20% tributyrin. Diets were fed in two phases (14 and 28 d). Pigs were confirmed positive for rotavirus A, B, and C and F18 enterotoxigenic *Escherichia coli* (*E. coli)*. There was no effect of dietary fiber or tributyrin on the proportion of pigs with normal, soft, or liquid fecal consistency in wk 1, 2, or 3 of the study (*P *> 0.10). There were no significant differences in average daily gain, average daily feed intake (ADFI), or gain-to-feed ratio (G: F) among treatments in phase 1 (*P *> 0.10). Tributyrin tended to increase ADFI in phase 2 compared to CON and BP (*P *= 0.063), and increased ADFI overall compared to CON (*P *= 0.031), but had no significant effect on final body weight (*P *> 0.10). Dietary treatment did not affect ileal, colonic, or fecal VFA concentrations on d 7, 10, or 42. However, on d 22, CON pigs had greater fecal acetate, propionate, valerate, and total VFA concentrations than one or more fiber treatments (*P *< 0.05), with butyrate tending to be greater than WB (*P *= 0.080). Bacterial community analysis revealed no effect of dietary fiber treatment on alpha diversity, except for DDGS, where d 14 and d 42 communities differed (*P *= 0.014). Beta-diversity analysis identified distinct clustering between time points (*P *= 0.001) and between treatments (*P *= 0.024) using the Bray Curtis dissimilarity matrix. Overall, although bacterial community composition was affected, the inclusion of WB, BP, corn DDGS, or tributyrin did not significantly impact intestinal health, pathogen load, VFA concentration, or growth performance in nursery pigs.

## Teaser

Dietary fiber and volatile fatty acids are proposed to improve gut health and nursery pig growth performance. However, under natural enteric disease challenge conditions, these strategies did not enhance growth performance or intestinal health despite altering gut microbial composition.

## Introduction

The weaning transition is often accompanied by reduced feed intake, compromised growth performance, and increased diarrheal incidence, commonly known as post-weaning diarrhea (PWD) (Lallès et al. 2007). During this period, pigs are susceptible to enteric pathogens that can exacerbate PWD, such as post-weaning colibacillosis caused by enterotoxigenic *Escherichia coli* (ETEC) (Hampson and Pluske 2004). Although therapeutic and subtherapeutic doses of antibiotics have been routinely used to mitigate enteric pathogen disturbances and enhance the performance of nursery pigs, alternatives to antibiotics are of interest as a strategy to manage enteric disease and maintain productivity (Gallois et al. 2009). Research has demonstrated that dietary fiber and volatile fatty acids (VFA) can be used to mitigate the incidence and severity of pathogens associated with PWD and improve pig health and performance (Whitney et al. 2006; Molist et al. 2009, 2010; Li et al. 2020).

There are various methods to classify dietary fiber. The detergent method can extract neutral detergent fiber (NDF), recovering the insoluble components of dietary fiber such as cellulose, hemicellulose, and lignin (Agyekum and Nyachoti 2017). The NDF procedure can accurately approximate the insoluble dietary fiber fraction of feed, however, it does not recover soluble dietary fiber. Therefore, the total dietary fiber (TDF) method can be used to separate dietary fiber into soluble and insoluble fractions. In pigs, insoluble fiber sources have been found to decrease digesta transit time, thereby reducing pathogen proliferation in the gastrointestinal tract (Wenk 2001; Molist et al. 2014; Jha and Berrocoso 2015). Insoluble fiber is resistant to fermentation and generates fewer VFA compared to soluble fiber, while also increasing fecal dry matter and bulkiness (Jha and Berrocoso 2015; Batson et al. 2021). Soluble fiber sources have a higher water-holding capacity and a large surface area for microbial activity, making them rapidly fermentable by microbes in the distal small intestine and large intestine (Jha and Berrocoso 2015). Altogether, dietary fiber has emerged as a potential strategy to mitigate the incidence and severity of pathogens associated with PWD by promoting the growth of a beneficial bacterial community that prevents the colonization of opportunistic pathogens (Kim et al. 2012; Laitat et al. 2015), decreasing expression of clinical signs (Bilic and Bilkei 2003), and increasing hindgut fermentation (Molist et al. 2009).

The gut microbiota in pigs consists of a diverse community of microorganisms, including bacteria, fungi, protozoa, and archaea, that interact with each other and with the host to influence biological processes vital for host health (Aluthge et al. 2019). The gut microbiome plays key roles in fiber fermentation, immune system maturation, and protection against harmful bacteria (Koh et al. 2016; Makki et al. 2018). Gut-associated bacterial communities are essential for fiber fermentation in the gastrointestinal tract, generating VFAs, including acetate, butyrate, and propionate, as well as hydrogen, carbon dioxide, and methane (Williams et al. 2001). These VFAs are absorbed by the colonic mucosa, with acetate entering systemic circulation via the liver for metabolism by muscle tissue, and butyrate being preferentially metabolized by colonocytes, contributing to approximately 70% of the total energy consumption of the colon (Williams et al. 2001). Butyrate supplementation in pigs has been shown to have beneficial effects on growth, feed intake, intestinal morphology, and reductions in *E. coli* in the gastrointestinal tract (Gálfi and Bokori 1990; Gálfi et al. 1996). These findings underscore the significant role of VFAs, particularly butyrate, in promoting growth performance and intestinal maturation and health in pigs.

Dietary fiber is increasingly recognized as a nutritional strategy to improve intestinal health and resilience in nursery pigs; however, its benefits are highly dependent on fiber source and inclusion level. Therefore, this study evaluated the effects of dietary fiber sources and tributyrin on intestinal health, bacterial community composition, VFA concentration, and growth performance in nursery pigs. It was hypothesized that inclusion of both insoluble and soluble fiber sources would shift the bacterial community toward beneficial taxa such as *Lactobacillus*, which supports enteric health and improved growth performance in nursery pigs. Further, it was hypothesized that increasing VFA availability in the small intestine via tributyrin supplementation would enhance small intestinal health and pig performance.

## Materials and methods

This study was conducted under a protocol approved by the Iowa State University Institutional Animal Care and Use Committee (IACUC protocol #23-152), in accordance with institutional standards for the humane treatment of research animals.

### Experimental design, housing, and dietary treatments

This study was conducted in two replicates, a total of 610 freshly weaned (19–21 d of age), mixed-sex pigs (5.60 ± 0.60 kg body weight [BW]; Camborough 1050 × 337 PIC, Hendersonville, TN) from a sow-nursery flow with a history of rotavirus and ETEC, were obtained from a commercial swine farm and shipped approximately 5 h to Ames, IA. In replicate 1, 160 pigs were allocated across 40 pens to obtain 4 pigs/pen. In replicate 2, 450 pigs were allocated across 45 pens to obtain 10 pigs/pen. Pens were randomly assigned to one of five dietary treatments (*n* = 17 pens/treatment). In both replicates, the study duration was 42 d and diets were fed in two phases consisting of 14 and 28 d, respectively.

Phase 1 (Table 1) and phase 2 (Table 2), diets were formulated to be isocaloric, isolysogenous, and were formulated to meet or exceed dietary minimums as reported in the NRC (2012) for the body weight of pigs used in this study. The five dietary treatments consisted of the following: 1) control diet (CON), 2) coarse wheat bran (WB), 3) beet pulp (BP), 4) corn distillers dried grains with solubles (DDGS), and 5) CON plus 0.20% tributyrin (TB: Prophorce™ SR130, Perstorp, NH Waspik, the Netherlands). Compared to the CON diet, in both diet phases, WB, BP, and DDGS were formulated to increase NDF by 30%. The TB product delivers butyric acid as a triglyceride, allowing it to survive gastric acidity and be hydrolyzed by pancreatic enzymes in the small intestine. All diets and water were provided ad libitum throughout the study.

**Table 1:** Phase 1 diets, as fed.

Ingredient (%)	Control	Wheat bran	Beet pulp	DDGS	Tributyrin
**Corn**	53.48	47.61	47.66	47.37	53.27
**Soybean meal, 46.5% CP**	20.00	20.00	20.00	20.00	20.00
**Soybean oil**	0.13	1.24	0.92	0.17	0.12
**Whey, dried**	13.89	13.89	13.89	13.89	13.89
**Limestone**	0.53	0.57	0.43	0.68	0.53
**Monocalcium phosphate 21%**	0.14	0.09	0.12	0.10	0.14
**Salt**	0.50	0.50	0.50	0.50	0.50
**L-lysine HCl**	0.34	0.34	0.33	0.36	0.34
**DL-methionine**	0.17	0.17	0.19	0.16	0.17
**L-threonine**	0.13	0.13	0.13	0.12	0.13
**L-tryptophan**	0.04	0.03	0.04	0.04	0.04
**L-valine**	0.03	0.02	0.03	—	0.03
**Vitamin Premix^1^**	0.23	0.23	0.20	0.23	0.23
**Trace Mineral Premix^2^**	0.20	0.20	0.20	0.20	0.20
**Fish meal, menhaden**	5.16	4.95	5.33	4.20	5.18
**Hamlet Protein 300**	5.00	5.00	5.00	5.00	5.00
**Phytase^3^**	0.03	0.03	0.03	0.03	0.03
**Wheat bran**	—	5.00	—	—	—
**Beet pulp**	—	—	5.00	—	—
**Corn DDGS^4^**	—	—	—	6.95	—
**Tributyrin^5^**	—	—	—	—	0.20
**Calculated composition**					
** Dry matter (%)**	90.51	90.62	90.58	90.00	90.50
** ME (kcal/kg)^6^**	3,351	3,351	3,351	3,351	3,351
** Crude protein (%)**	23.09	23.32	23.13	23.70	23.09
** Acid detergent fiber (%)**	2.74	3.20	3.69	3.45	2.74
** Neutral detergent fiber (%)**	5.44	7.09	7.09	7.09	5.43
** Insoluble fiber (%)**	5.96	5.62	6.87	5.61	5.96
** Soluble fiber (%)**	1.06	1.03	1.99	1.03	1.06
** Total calcium (%)**	0.68	0.68	0.68	0.68	0.68
** Available phosphorus (%)**	0.48	0.48	0.48	0.48	0.48
** SID Lysine (%)^7^**	1.40	1.40	1.40	1.40	1.40

1 Diet was provided with 6,125 IU vitamin A, 700 IU vitamin D3, 50 IU vitamin E, 30 mg vitamin K, 0.05 mg vitamin B12, 11 mg riboflavin, 56 mg niacin, and 27 mg pantothenic acid per kilogram of diet.

2 Diet was provided with 9 mg Cu (as CuSO4), 120 mg Fe (as FeSO4), 0.21 mg I (as Ca(IO3)2), 6.75 mg Mn (as MnSO4), 120 mg Zn (as ZnSO4), and 0.23 mg Se (as Na2SeO3) per kilogram of diet.

3 Huvepharma, Peachtree City, GA, USA. Minimum activity of 750 phytase units (FTU)/kg complete feed.

4 DDGS = corn distiller’s dried grains with soluble.

5 Perstorp, NH Waspik, The Netherlands. ProPhorce™ SR 130 (2 kg/MT).

6 ME, metabolizable energy.

7 Standardized ileal digestible lysine.

**Table 2:** Phase 2 diets, as fed.

Ingredient (%)	Control	Wheat bran	Beet pulp	DDGS	Tributyrin
**Corn**	59.22	52.62	52.82	54.84	58.99
**Soybean meal, 46.5% CP**	37.04	36.43	36.5	33.31	37.08
**Soybean oil**	0.33	1.61	1.31	0.17	0.32
**Limestone**	0.95	0.98	0.84	1.06	0.95
**Monocalcium phosphate 21%**	0.85	0.75	0.86	0.64	0.85
**Salt**	0.50	0.50	0.50	0.50	0.50
**L-lysine HCl**	0.35	0.35	0.35	0.42	0.35
**DL-methionine**	0.16	0.16	0.18	0.15	0.16
**L-threonine**	0.12	0.12	0.14	0.13	0.12
**L-tryptophan**	0.02	0.02	0.02	0.03	0.02
**L-valine**	—	—	0.02	—	—
**Vitamin Premix^1^**	0.23	0.23	0.23	0.23	0.23
**Trace Mineral Premix^2^**	0.20	0.20	0.20	0.20	0.20
**Phytase^3^**	0.03	0.03	0.03	0.03	0.03
**Wheat bran**	—	6.00	—	—	—
**Beet pulp**	—	—	6.00	—	—
**Corn DDGS^4^**	—	—	—	8.29	—
**Trubutyrin^5^**	—	—	—	—	0.20
**Calculated composition**					
** Dry matter (%)**	88.97	89.11	89.07	88.87	88.99
** ME (kcal/kg)^6^**	3,307	3,307	3,307	3,307	3,307
** Crude protein (%)**	21.92	22.10	22.00	22.03	22.00
** Acid detergent fiber (%)**	4.00	4.30	5.00	4.45	4.00
** Neutral detergent fiber (%)**	6.60	8.54	8.55	8.55	7.00
** Insoluble fiber (%)**	8.76	8.29	9.80	7.97	8.76
** Soluble fiber (%)**	1.74	1.68	2.84	1.57	1.74
** Total calcium (%)**	0.65	0.65	0.65	0.65	0.65
** Available phosphorus (%)**	0.37	0.37	0.37	0.37	0.37
** SID Lysine (%)^7^**	1.35	1.35	1.35	1.35	1.35

1 Diet was provided with 6,125 IU vitamin A, 700 IU vitamin D3, 50 IU vitamin E, 30 mg vitamin K, 0.05 mg vitamin B12, 11 mg riboflavin, 56 mg niacin, and 27 mg pantothenic acid per kilogram of diet.

2 Diet was provided with 9 mg Cu (as CuSO4), 120 mg Fe (as FeSO4), 0.21 mg I (as Ca(IO3)2), 6.75 mg Mn (as MnSO4), 120 mg Zn (as ZnSO4), and 0.23 mg Se (as Na2SeO3) per kilogram of diet.

3 Huvepharma, Peachtree City, GA, USA. Minimum activity of 750 phytase units (FTU)/kg complete feed.

4 DDGS = corn distiller’s dried grains with soluble.

5 Perstorp, NH Waspik, The Netherlands. ProPhorce™ SR 130 (2 kg/MT).

6 ME, metabolizable energy.

7 Standardized ileal digestible lysine.

Individual pig BW, feed and feeder weights were recorded on d 0, 7, 14, 28, and 42 and utilized to calculate feed disappearance, pen average daily gain (ADG), average daily feed intake (ADFI), and Gain:Feed (G:F). Pen fecal consistency scores were evaluated daily from d 0 to 21 and three times weekly for the remainder of the study by the same individual. The fecal scoring rubric used a subjective scale of 0 to 3, with 0 representing solid, dry stool, 1 representing semi-solid, 2 representing soft, and 3 representing a liquid fecal consistency in the pens. Removals and mortalities were recorded.

### Sample collection

On d 7 post-placement, fresh fecal samples were collected from pens, pooled, and sent to Iowa State’s Veterinary Diagnostic Laboratory in Ames, IA for population-level rotavirus detection by real-time PCR (RT-PCR) and aerobic culture for hemolytic *E. coli*. To further assess early post-weaning enteric health, VFA concentrations, and intestinal morphology, in replicate 1, one pig of median body weight per pen (*n* = 8/treatment) was selected and euthanized by captive bolt and subsequent exsanguination on d 7. Ileal and colonic contents were collected for VFA analysis, and ileal pH was measured using plastic pH indicator strips ranging from 5.0 to 9.0 (ThermoFisher Scientific, Waltham, MA, USA). Sections of distal ileum 40 cm from the ileocecal junction and apex of the colon were collected into 10% neutral buffered formalin. After 24 h, fixed samples were transferred to 70% ethanol and were sent to the Iowa State Veterinary Diagnostic Laboratory (Ames, IA) where they were paraffin-embedded, sectioned, mounted onto slides, and stained for histological evaluation. Fecal samples were collected from replicate 2 pigs on d 10, 22, and 42 and pooled by pen for VFA analysis. In addition, pooled fecal samples were collected from all pens on d 7, 14, 23, and 42 for F18 and LT gene abundance via RT-PCR and microbial community analysis. For F18 and LT gene abundance on d 7, 23, and 42, five collected pens per treatment were randomly selected for RT-PCR analysis (2 pens from replicate 1 and 3 pens from replicate 2). On d 14, 9 collected pens per treatment were randomly selected for RT-PCR analysis (4 pens from replicate 1 and 5 pens from replicate 2).

### Intestinal morphology

Sections of the distal ileum and colon were stained with hematoxylin and eosin to evaluate intestinal morphology as previously described by Nisley et al. (2026). Images were captured on an Olympus BX53/43 motorized-stage microscope fitted with a DP80 digital camera (Olympus Scientific, Waltham, MA). Villus and crypt measurements were taken digitally with CellSens Dimension software, version 1.16 (Olympus Scientific, Waltham, MA). Measurements of ileum villus height (µm) and crypt depth (µm) were used to determine villus-to-crypt ratios. In colonic sections, only crypt depth was measured (µm).

### Fecal F18 and LT gene abundance

Fecal F18 and LT gene abundance were determined as previously described (Due et al. 2025). RT-PCR reactions were performed with TaqMan Fast Advanced Master Mix (ThermoFisher Scientific, Waltham, MA, USA) using 20× gene-specific primers for *F18* and *LT*. The sequences for the *F18* and *LT* primer sets were as follows: *F18* forward primer (5’-3’): GCGTCGAATAGCACTGTAAGTTTC, *F18* reverse primer: ACCACCTTTCAGTTGAGCAGTAAAT, *LT* forward primer (5’-3’): GCAGGCAAAAGAGAAATGGTTATCA, and *LT* reverse primer: CCGGGACTTCGACCTGAA (ThermoFisher Scientific, Waltham, MA, USA).

Each 20 µL fecal sample reaction contained 10 µL of TaqMan Fast Advanced Master Mix, 1 µL of F18 primer, 1 µL of LT primer, 1 µL of template DNA (50 ng/µL, 260/280 ratio of 1.8–2.0), and 7 µL of nuclease-free water. Fluorescence was quantified using a QuantStudio^TM^ 3 RT-PCR System (ThermoFisher Scientific, Waltham, MA, USA) with the following conditions: 20-s hold for polymerase activation at 95°C followed by 40 RT-PCR cycles (denature at 95°C for 1 s, anneal/extend at 60°C for 20 s). QuantStudio^TM^ Design and Analysis Software (ThermoFisher Scientific, Waltham, MA, USA) was used to analyze the amplification plot, and cycle threshold (Ct) values for each reaction were recorded. Data were reported as an adjusted Ct value, calculated as the difference between the determined Ct value of the gene of interest and the maximum Ct value at 40 amplification cycles (Due et al. 2025).

### VFA analysis

Ileal and colonic contents were collected on d 7 from 8 pigs/treatment, and pen-pooled fecal samples were collected on d 10, 22, and 42. The VFA content of samples was analyzed by Gas chromatography–mass spectrometry (GC-MS) analysis at the Iowa State University W.M. Keck Metabolomics Research Laboratory, Ames, IA, as previously described by Miller et al. (2025), utilizing a method adapted from Hsu et al. (2019). Briefly, previously frozen samples were thawed on ice, and 300 mg of each sample was transferred and weighed into microcentrifuge tubes. Samples were spiked with an internal standard before being acidified and homogenized with metal beads and subjected to a liquid–liquid extraction process using 1-butanol to extract VFAs. Following phase separation, the upper butanol layer containing the VFAs was collected for subsequent GC-MS analysis. The VFA standards were prepared and analyzed using the same method to serve as references. VFA analysis was conducted using an Agilent 7890C gas chromatograph coupled to a 5975C Mass Spectrometer equipped with an Agilent 19091F-433 HP-FFAP column (30 m × 250 μm × 0.25 μm). 1 µL of the butanol VFA extract was injected under split inlet mode with a 6:1 split. The inlet and transfer line were each held at 240°C and the system operated with helium carrier gas at a flow rate of 1 mL/min. The gas chromatograph oven program began at 50°C which was held for 1 min and was ramped linearly at a 25°C/min to 200°C before heating at 10°C/min to a final temperature of 240°C. The mass spectrometer operated under standard conditions with a 230°C ion source and a mass acquisition range of m/z 35–450. Data analysis was completed using Agilent MassHunter Qualitative and Quantitative Analysis programs (versions 10.0). Identification and quantification were conducted primarily using corresponding VFA standards and the acetate-d3 internal standard, with additional identification performed using the NIST20 and Wiley 11 GC-MS spectral libraries. The VFA concentrations were reported as µmoles VFA/g feces on a as is (wet) basis.

### Bacterial community analysis

Pooled pen fecal samples were collected on d 14 and 42 from 45 pens (*n* = 9/treatment) for microbial community analysis. DNA was extracted from 90 fecal samples and 3 negative control samples (kit controls) using the Mag-Bind Universal Pathogen 96 kit (Omega Bio-Tek Inc., Norcross, GA) according to the manufacturer’s protocol. Negative controls were prepared by substituting 250 μL of nuclease-free water for fecal samples to monitor for kit contamination. Briefly, samples were combined with 275 μL of SLX-Mlus buffer in 2-mL safe-lock tubes and subjected to mechanical lysis using a TissueLyser (QIAGEN Inc., Valencia, CA) at 20 Hz for 10 min. An additional lysis step was performed by incubating the samples at 90°C for 10 min. The resulting samples were then centrifuged at 3,500 × g for 10 min, and the supernatant was transferred to a 96-well deep plate. The final purification of the DNA was performed using a KingFisher purification system (ThermoFisher Scientific, Waltham, MA, USA). The elution buffer was heated at 70°C to increase DNA yield. After DNA extraction, 5 μL of the resulting DNA was subjected to gel electrophoresis on a 1.5% agarose gel at 120 V for 40 min to assess DNA quality, and samples were stored at −20°C until use for PCR amplification and sequencing.

The V4 region of the 16S rRNA gene was amplified using dual-indexed PCR primers as described by Kozich et al. (2013). Each 25 μL PCR reaction contained 1× Terra PCR Direct Polymerase kit (Takara Bio USA Inc., San Jose, CA), 0.625 units of Terra Taq polymerase, 0.4 μM of each dual-indexed 16S rRNA forward and reverse primers, and 2.0 μL of DNA (5–20 ng). DNA amplification was performed on a Veriti 96-Well Thermal Cycler (Life Technologies, Carlsbad, CA) with the following conditions: 98°C for 3 min; 25 cycles of 98°C for 30 s, 55°C for 30 s, and 68°C for 45 s; followed by a final extension at 68°C for 4 min. The PCR products were visualized on a 1.5% agarose gel to verify DNA fragment size (∼400 bp). In addition to PCR negatives, DNA negative control samples were also processed to ensure no contamination from reagents, and was confirmed by no amplification upon gel electrophoresis. PCR products were normalized to the same concentration using NGS Normalization 96-Well Kit (Norgen BioTek Corp., Thorold, ON, Canada) following manufacturer’s instructions. Normalized products were then pooled and analyzed on an Agilent Bioanalyzer 2100 using the High Sensitivity DNA chip (Agilent Technologies, Santa Clara, CA) to verify the library fragment size (∼400 bp). Quantification of the libraries was performed through real-time PCR to measure pooled library concentration for sequencing. The libraries were diluted to 2 nM and sequenced using a V2, 500 cycle kit on the Illumina MiSeq platform (llumina Inc., San Diego, CA) according to the manufacturer’s protocol.

### Bioinformatic analysis of fecal bacterial communities

All bioinformatic data processing and analysis steps were conducted in R (Version 4.4.2). Briefly, raw FASTQ files were processed using the DADA2 pipeline (Callahan et al. 2016) with default parameters. Reads were filtered to remove low-quality reads and, any reads with a Q score of less than 30 bp were removed. The error rates were learned and high-quality forward and reverse reads were assembled to generate contigs that represent the 250 bp V4 region sequenced. Reads were binned to identify amplicon sequence variants (ASVs) following chimera removal, and singleton ASVs were excluded from the dataset. Taxonomy was assigned to ASVs using the Silva reference database (Silva version 138.2), compatible with DADA2 pipeline. Mothur v.1.35.1 (Schloss et al. 2009) was used to generate a phylogenetic tree. The resulting phylogenetic tree, taxa table, ASVs table, and mapping file were merged to generate a “phyloseq object” for further analysis using phyloseq package version 1.50.0 (McMurdie and Holmes 2013). Only ASVs present in at least two samples with a minimum abundance of 0.15% were kept in the dataset (Tom et al. 2024). The sequenced negative control were used to remove any contaminants that may have resulted from the extraction and PCR reagents using the decontam package version 1.26.0 (Davis et al. 2018). Bacterial richness (alpha-diversity) was assessed using the Shannon index and observed ASVs. All reads per sample were rarefied to the uniform depth before estimating alpha diversity.

Wilcoxon test was used to assess the differences between time points (d 14 vs. d 42) and between treatment groups (CON, WB, BP, DDGS, TB) in alpha-diversity. Beta-diversity was performed using unweighted UniFrac distance and Bray-Curtis dissimilarity matrix to measure microbial community composition differences across days and treatments. Permutational multivariate analysis of variance (PERMANOVA) was used to assess differences in bacterial community composition between treatment groups using the adonis function in the vegan package version 2.6.8 (Dixon 2003).

The model used for the PERMANOVA included the main effects of treatment (CON, WB, BP, DDGS, TB) and time-point (d 14 and d 42). Differentially abundant ASVs between treatment groups were also identified using DESeq2 Version 1.46.0 using Benjamini-Hochberg false discovery adjustment (Anders and Huber 2010).

### Statistical analysis

Statistical analyses were performed with SAS v. 9.4 (SAS Institute, Cary, NC), using the MIXED and GLIMMIX procedures as appropriate for the outcome measure. Performance data were evaluated within phase and overall (d 0–14, 14–42, and 0–42) for the main effect of treatment, and replicate was included as a random effect. Morphology, gene abundance, and VFA data were analyzed with treatment as the fixed effect and replicate as a random effect. For VFA data, the interaction between treatment and day was also assessed. Least square means were generated using the LSMEANS statement and are reported with a pooled standard error. Ordinal fecal consistency scores were analyzed using the GLIMMIX procedure, with pen treated as a repeated effect and replicate included as a random effect. For microbiome data, alpha diversity measures were compared using the Wilcoxon rank-sum test. Beta-diversity used PERMANOVA to assess differences in bacterial community composition between treatment groups. *P*-values were calculated using Chi-square tests for type 3 generalized estimating equations. Effects were declared significant when *P *≤ 0.05 and reported as a tendency when *P *≤ 0.10.

## Results

### Pig health

Within the first 7 d of placement, both replicate pig populations were confirmed positive for rotavirus and hemolytic *E. coli* via routine surveillance. Irrespective of diet, pig feces tested positive for rotavirus A (Ct = 10–16), B (Ct = 21–32), and C (Ct = 18–24) 1 wk post-weaning. These fecal samples also tested positive for hemolytic *E. coli* with high to heavy growth, and the *E. coli* isolates were genotyped and determined positive for the F18 fimbrial gene and the EAST1, LT, STb, Stx2, and Stx2e toxin genes. Assessment of PWD over the study duration is reported in Figure 1. Dietary fiber treatments or tributyrin did not impact the proportion of pigs with normal, soft, or liquid fecal consistency in wk 1, 2, or 3 of the study (*P *> 0.10). Over the last 21 d of the study, DDGS tended to result in a higher proportion of pigs with loose fecal consistency compared to WB, BP, and TB, while TB tended to result in a higher proportion of pigs with solid fecal scores compared to CON (*P *= 0.052).

**Figure 1:**
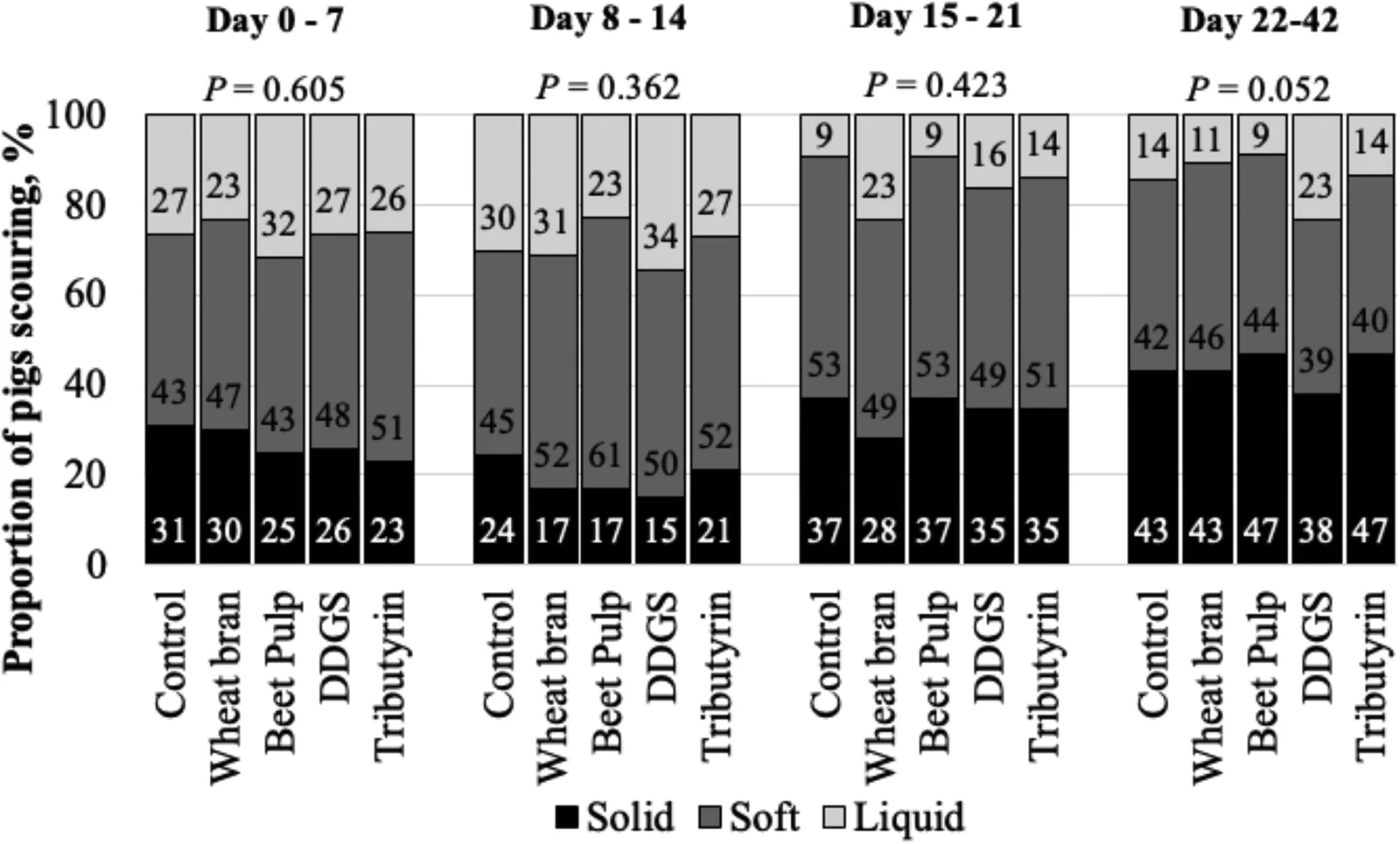
Pen fecal consistency scores were evaluated daily from d 0 to 21 and three times weekly for the remainder of the study to calculate the proportion of pens with solid (0-1), soft (2), or liquid (3) fecal consistency between dietary treatments (Control, Beet Pulp, DDGS, Tributyrin, and Wheat Bran). *N* = 17 pens/treatment.

Given that pigs were naturally challenged with hemolytic *E. coli* confirmed to be F18 fimbria and LT gene positive, fecal *F18* and *LT* gene abundance was assessed on d 7, 14, 23, and 42 to further evaluate the effects of dietary fiber and VFA supplementation on enteric health (Table 3). An adjusted Ct value was calculated as the difference between the determined Ct value of the gene of interest and the maximum Ct value of 40 amplification cycles. On d 7, 14, 23, and 42, *F18* and *LT* fecal adjusted Ct values were not different among treatments (*P *> 0.10). On d 7, 23, and 42, *F18* and *LT* fecal adjusted Ct values remained low, indicating low gene abundance. On d 14, as scouring increased, F18 and LT fecal adjusted Ct values increased, indicating elevated gene abundance and greater F18 ETEC burden. The subsequent decline in F18 and LT fecal gene abundance from d 14 onward is indicative of F18 ETEC clearance and resolution.

**Table 3:** Relative fecal F18 and LT toxin genes abundance in nursery pigs fed varying fiber sources or tributyrin.

Item	Treatment					SEM	*P*-value
	Control	Wheat bran	Beet pulp	DDGS	Tributyrin		
**Day 7^1^**							
** F18, adjusted Ct**	9.7	6.9	8.7	7.2	5.8	2.54	0.829
** LT, adjusted Ct**	6.9	4.3	7.3	5.1	4.2	2.39	0.818
**Day 14^2^**							
** F18, adjusted Ct**	13.7	17.1	17.1	19.0	14.2	1.89	0.262
** LT, adjusted Ct**	13.3	16.7	14.8	18.3	13.6	2.00	0.353
**Day 23^1^**							
** F18, adjusted Ct**	7.2	7.6	7.9	7.2	12.3	1.83	0.255
** LT, adjusted Ct**	5.0	5.0	5.4	4.8	9.6	1.72	0.269
**Day 42^1^**							
** F18, adjusted Ct**	5.4	5.3	5.4	5.6	6.0	0.21	0.149
** LT, adjusted Ct**	0.0	0.0	0.0	0.0	0.0	0.00	—

1 *n* = 5 pens/treatment

2 *n* = 9 pens/treatment

Means reported as adjusted Ct, or the difference in Ct value for gene of interest and maximum Ct value of 40 amplification cycles.

### Growth performance

Pig body weights at d 7, 14, and 42 did not differ across treatments (*P *> 0.10; Table 4), although WB, BP, DDGS, and TB were ∼10% higher than the CON by d 42. There was no impact of dietary fiber or tributyrin on ADG, ADFI and G:F in phase 1 (*P *> 0.10). There was no overall (d 0–42) difference in ADG and G:F among dietary treatments (*P *> 0.10). Similarly, in phase 2 (d 15–42), ADG and G:F were not different (*P *> 0.10). Compared to CON and BP, ADFI tended to be increased by 14% in TB, with WB and DDGS in between (*P *= 0.063). Removal over the 42-d study tended to be lowest in DDGS (3%) compared to WB (13%), CON (11%), TB (8%), and BP (7%) dietary treatments (*P *= 0.098). Overall ADFI was 13% higher in TB compared to CON, with WB, BP, and DDGS overall ADFI being intermediate (*P *= 0.031**)**.

**Table 4:** Growth performance of pigs fed varying fiber sources or tributyrin.

Item	Treatment**^1^**					SEM	*P*-value
	Control	Wheat bran	Beet pulp	DDGS	Tributyrin		
**Body weight (kg)**							
** Day 0**	5.5	5.8	5.5	5.7	5.6	0.26	0.724
** Day 7**	5.9	6.4	6.2	6.2	6.1	0.18	0.525
** Day 14**	6.9	7.2	7.4	7.3	7.3	0.22	0.454
** Day 42**	20.4	22.0	21.9	21.9	22.6	1.59	0.195
**Phase 1 (Day 0–14)**							
** ADG (kg/d)**	0.09	0.09	0.13	0.11	0.10	0.022	0.174
** ADFI (kg/d)**	0.17	0.18	0.20	0.19	0.19	0.023	0.300
** Gain:Feed**	0.48	0.51	0.64	0.59	0.50	0.070	0.396
**Phase 2 (Day 14–42)**							
** ADG (kg/d)**	0.55	0.58	0.58	0.60	0.63	0.037	0.323
** ADFI (kg/d)**	0.88^y^	0.93^x,y^	0.88^y^	0.93^x,y^	1.01^x^	0.090	0.063
** Gain:Feed**	0.63	0.64	0.65	0.65	0.62	0.036	0.873
**Overall (Day 0–42)**							
** ADG (kg/d)**	0.33	0.35	0.37	0.38	0.39	0.032	0.157
** ADFI (kg/d)**	0.63^b^	0.65^a,b^	0.64^a,b^	0.67^a,b^	0.71^a^	0.020	0.031
** Gain:Feed**	0.53	0.54	0.57	0.57	0.55	0.048	0.748
** Removals (%)**	11.4^x,y^	12.5^x^	7.0^x,y^	2.8^y^	7.5^x,y^	2.91	0.098

1 *n* = 17 pens/treatment.

a,b Means with differing superscripts differ significantly at *P *≤ 0.05.

x,y Means with differing superscripts tend to differ at *P *≤ 0.10.

### Intestinal morphology

Dietary fiber and tributyrin had no effect on ileal villus height, crypt depth, or the villus-to-crypt ratio (*P *> 0.10; Table 5). Furthermore, crypt depth measurements at the apex of the spiral colon remained consistent across treatments (*P *> 0.10). Ileal pH (Table 5) tended to be 5% lower in TB compared to CON, WB and BP, with DDGS having the highest ileal pH (*P *= 0.060).

**Table 5:** Ileum pH, ileum and colon measured morphology of pigs fed varying fiber sources or tributyrin.

Item	Treatment**^1^**					SEM	*P*-value
	Control	Wheat bran	Beet pulp	DDGS	Tributyrin		
**Ileum pH**	6.4^x,y^	6.3^x,y^	6.3^x,y^	6.9^x^	6.1^y^	0.19	0.060
**Ileum villus height (μm)**	258	249	237	249	277	21.2	0.754
**Ileum crypt depth (μm)**	216	195	209	197	206	11.4	0.666
**Ileum villus:crypt ratio**	1.3	1.4	1.3	1.4	1.4	0.12	0.851
**Colon crypt depth (μm)**	402	396	408	368	415	20.5	0.552

1 *n* = 8 pigs/treatment.

x,y Means with differing superscripts tend to differ at *P *≤ 0.10.

### VFA production

Day 7 ileal and colonic contents and pooled fecal samples on d 10, 22, and 42 were assessed for VFA concentration (Table 6). No differences in acetic, butyric, propionic, valeric or total ileal and colonic VFA concentrations were observed among dietary treatments on d 7 (*P *> 0.10). Similar to d 7 luminal VFA concentrations, dietary treatment did not impact d 10 and 42 fecal VFA concentrations (*P *> 0.10). However, a significant day effect was evident for acetic acid (*P *= 0.002), propionic acid (*P *= 0.034), and total VFA concentration (*P *= 0.048), with concentrations increasing over time. Day 22 fecal sample acetic acid concentration were notably higher in CON compared to WB (95.8 vs. 70.9 μmoles/g, respectively, *P *= 0.028). Propionic acid concentrations were also elevated in CON compared to WB and BP (36.7 vs. 26.2 and 25.6 μmoles/g, respectively, *P *= 0.041). While fecal valeric acid was increased in CON relative to all other treatments (6.0 vs. 3.3, 3.9, 4.6, and 5.4 μmoles/g for WB, BP, DDGS, and TB, respectively, *P *= 0.047). Butyric acid tended to be increased in CON compared to WB (20.5 vs. 12.9 μmoles/g, respectively, *P *= 0.080). Day 22 total VFA concentrations accounting for the sum of acetic, butyric, propionic, isobutyric, isovaleric, valeric, isocaproic, caproic, and heptanoic acids was higher in CON, 166.6 μmoles/g, compared to WB, 117.5 μmoles/g (*P *= 0.021).

**Table 6:** Ileal, colonic, and fecal volatile fatty acid concentrations (μmoles/g) of pigs varying fiber sources or tributyrin.

Measure	Treatment					SEM	*P*-value
	Control	Wheat bran	Beet pulp	DDGS	Tributyrin		
**Ileal contents^1^, Day 7**							
** Acetic**	3.1	2.6	3.6	4.6	4.1	1.27	0.816
** Butyric**	0.0	0.0	0.1	0.1	0.2	0.08	0.515
** Propionic**	0.6	0.5	1.0	0.5	0.7	0.18	0.203
** Valeric**	0.3	0.3	0.3	0.2	0.2	0.03	0.172
** Total^3^**	4.3	3.7	5.5	5.8	5.6	1.43	0.797
**Colon contents^1^, Day 7**							
** Acetic**	34.8	34.0	34.2	28.8	36.6	3.77	0.673
** Butyric**	5.3	4.6	4.3	3.2	5.4	0.76	0.257
** Propionic**	12.4	10.2	10.8	10.0	16.4	1.99	0.149
** Valeric**	1.8	1.5	1.5	1.0	1.8	0.26	0.190
** Total^3^**	55.7	51.5	52.2	44.1	61.5	6.31	0.416
**Feces^2^, Day 10**							
** Acetic**	75.8	69.9	70.4	67.3	65.4	5.17	0.677
** Butyric**	17.5	15.1	12.3	11.0	11.3	3.21	0.561
** Propionic**	28.9	23.9	25.5	23.2	25.3	2.69	0.616
** Valeric**	6.3	4.2	3.0	2.4	3.5	1.37	0.325
** Total^3^**	136.9	119.6	115.1	106.6	109.2	13.44	0.538
**Feces^2^, Day 22**							
** Acetic**	95.8^a^	70.9^b^	79.4^a,b^	76.1^a,b^	81.3^a,b^	5.30	0.028
** Butyric**	20.5^x^	12.9^y^	16.3^x,y^	14.4^x,y^	15.9^x,y^	1.88	0.080
** Propionic**	36.7^a^	26.2^b^	25.6^b^	28.7^a,b^	33.0^a,b^	2.82	0.041
** Valeric**	6.0^x^	3.3^y^	3.9^y^	4.6^y^	5.4^y^	0.67	0.047
** Total^3^**	166.6^a^	117.5^b^	131.4^a,b^	128.6^a,b^	141.5^a,b^	10.20	0.021
**Feces^2^, Day 42**							
** Acetic**	81.7	75.9	80.7	87.0	75.5	4.93	0.468
** Butyric**	16.1	13.8	14.7	16.0	14.5	1.64	0.830
** Propionic**	31.5	28.3	29.3	31.8	32.4	2.26	0.673
** Valeric**	5.2	3.5	3.6	4.5	4.8	0.63	0.289
** Total^3^**	140.5	125.2	133.1	144.0	132.7	9.25	0.643

1 *n* = 8 pigs/treatment.

2 *n* = 8 pens/treatment.

3 Total VFAs represent the sum of acetic, butyric, propionic, isobutyric, isovaleric, valeric, isocaproic, caproic, and heptanoic acids. Fatty acid concentrations are reported in μmoles/g on an as is (wet) matter basis.

a,b Means with differing superscripts differ significantly at *P *≤ 0.05.

x,y Means with differing superscripts tend to differ at *P *≤ 0.10.

### Bacterial community analysis

In this study, 16S rRNA gene amplicon sequencing was used to investigate how soluble and insoluble dietary fiber types alter bacterial community composition in the gastrointestinal tract of pigs. After removing negative control samples and quality filtering, the dataset resulted in 90 samples with 2,901,564 reads (an average of 32,239 reads per sample), which were binned into 526 ASVs. Prior to alpha diversity analyses, reads were rarefied to a uniform depth of 6,154 reads per sample to eliminate the influence of read depth on bacterial diversity. Alpha diversity differences between d 14 and d 42 and among dietary fibers treatments were investigated (Figure 2). The Shannon diversity index and observed ASVs significantly increased at d 42 compared to d 14 (*P *= 0.003 and *P *< 0.001, respectively). However, there was no significant effect of dietary fiber treatment on the Shannon diversity or observed ASVs within each time point, except for DDGS when compared between d 14 and d 42 (*P *= 0.014). All treatment groups displayed greater representation of Bacillota at d 14 and 42 at the phylum level. At the genus level, *Lactobacillus* dominated fecal microbial communities at d 14; however, on d 42, this proportion decreased significantly (*P *< 0.001; Figure 3). Additionally, *Streptococcus* was lower in abundance on d 14 but increased significantly by d 42 (*P *< 0.001; Figure 3). Assessment of fecal *E. coli* abundance on d 14 and 42 is reported in Figure 4. The control and BP fed pigs had lower fecal *E*. coli abundances compared to WB (*P *< 0.05), with DDGS and tributyrin diets being intermediate.

**Figure 2:**
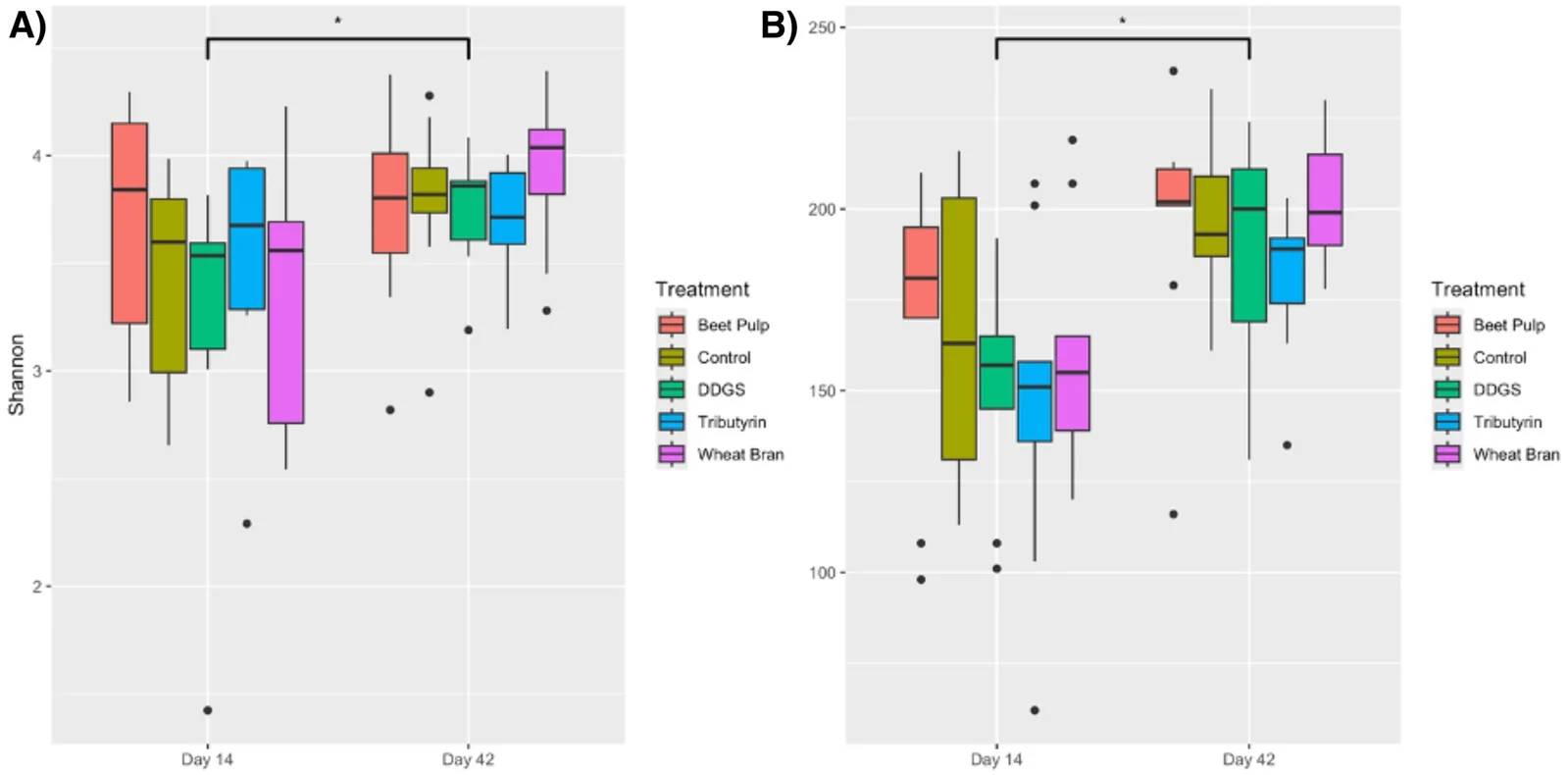
Pooled pen fecal samples were collected on d 14 and 42 post-weaning to evaluate differences in bacterial alpha diversity among dietary treatments (Control, Beet Pulp, DDGS, Tributyrin, and Wheat Bran) at each time point (D 14 and D 42). Box-and-whisker plots compare alpha diversity across treatments using the (A) Shannon index and (B) observed ASVs. Statistical comparisons were performed using the Wilcoxon rank-sum test. Boxes represent the interquartile range (25th–75th percentiles), with the median indicated by the horizontal line. Whiskers denote the minimum and maximum values, and outliers are shown as black dots and *P < 0.05.

**Figure 3:**
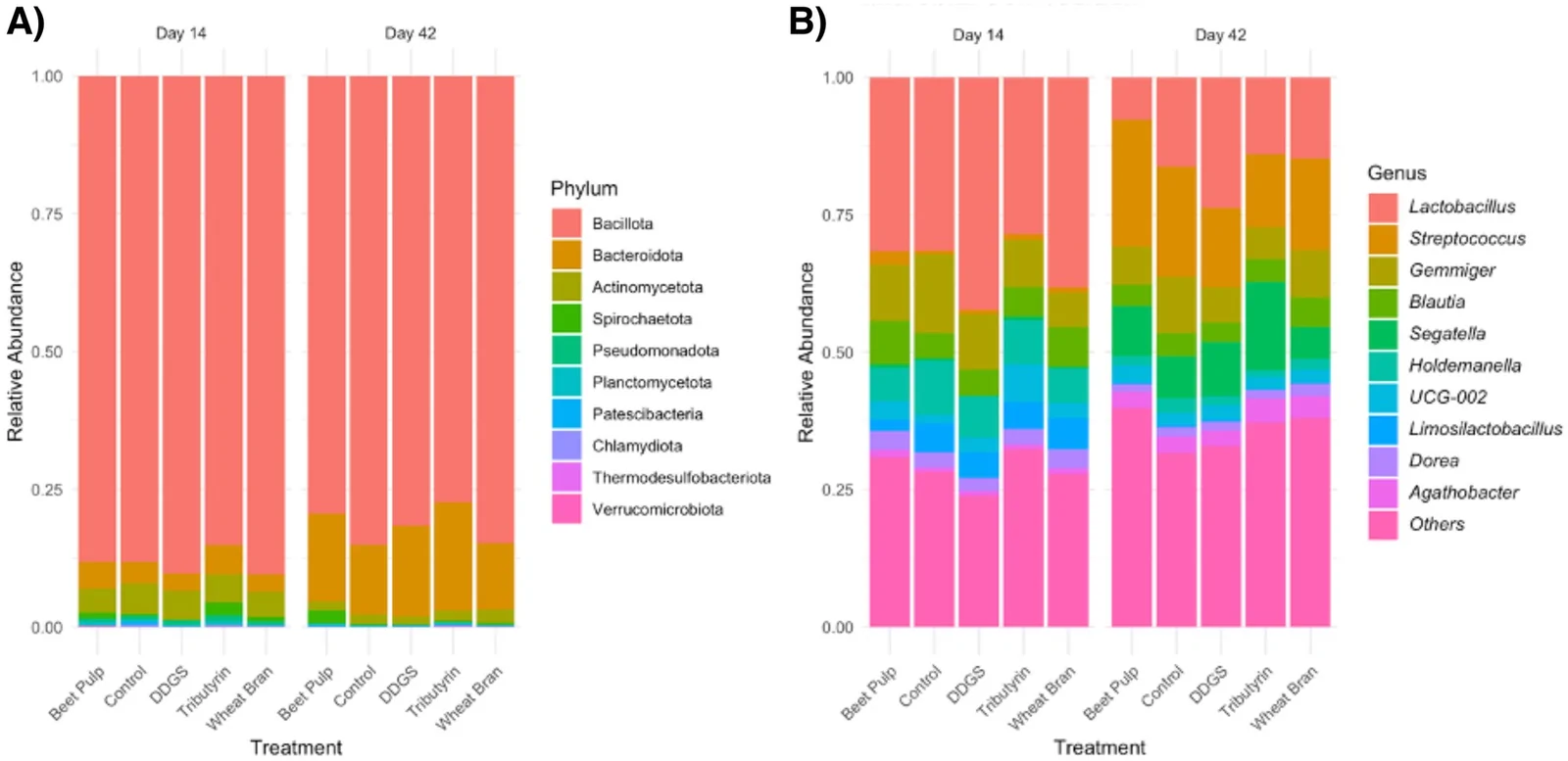
Fecal microbial community composition across time points (D 14 and D 42) and dietary treatments (Control, Beet Pulp, DDGS, Tributyrin, and Wheat Bran). Microbial community composition is shown at the (A) phylum level and (B) genus level (top 10 taxa). Each color in the stacked bar charts represents the relative abundance of bacterial taxa.

**Figure 4:**
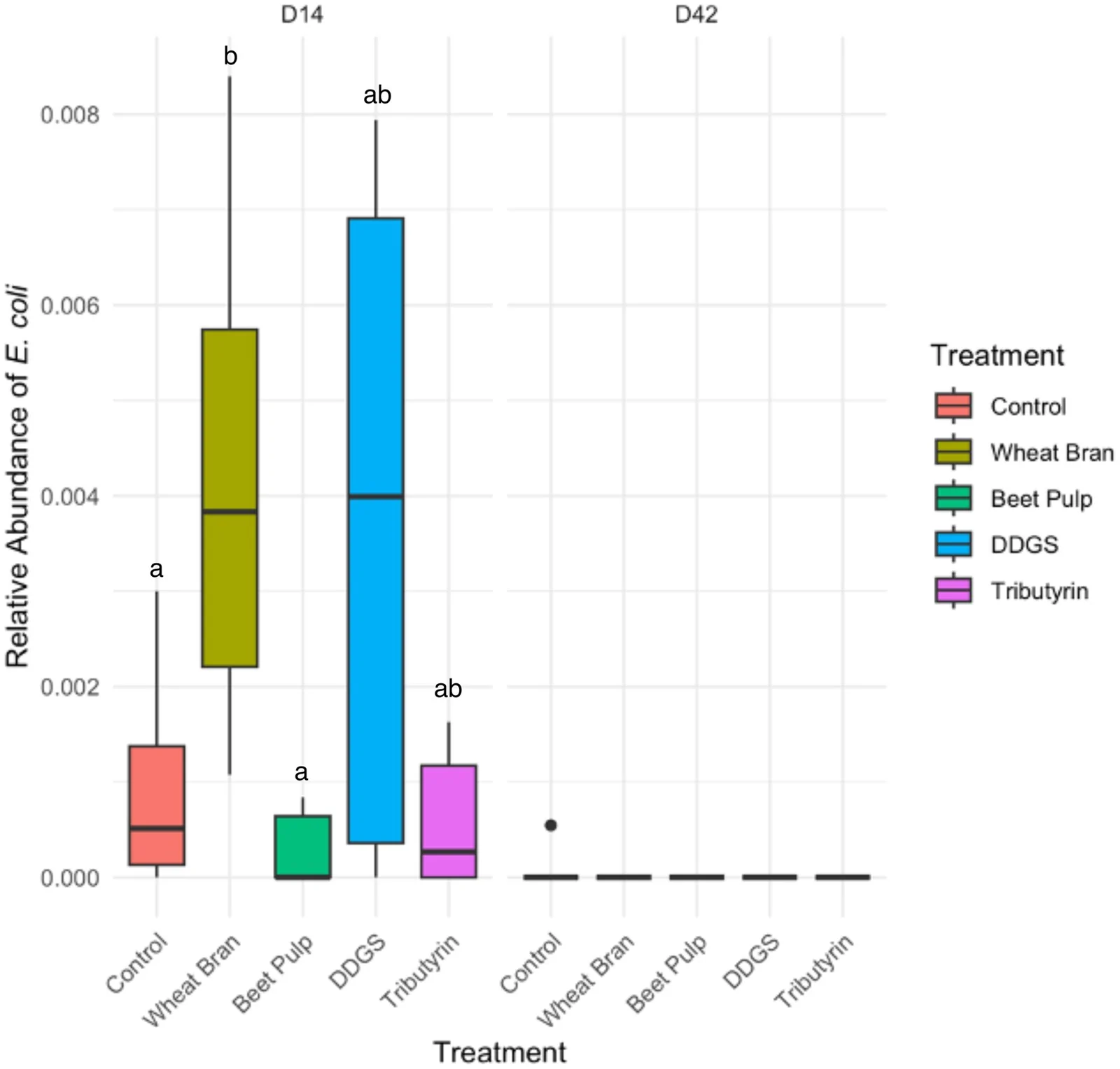
Relative abundance of fecal *E. coli* across time points (D 14 and D 42) and dietary treatments (Control, Beet Pulp, DDGS, Tributyrin, and Wheat Bran). Within day, different letters (a, b) represent *P *< 0.05.

To visualize the dynamic changes in relative abundance of microbial communities, beta-diversity analysis was performed using the Bray-Curtis dissimilarity matrix with PERMANOVA (Figure 5). This analysis revealed significant differences in bacterial community composition between time points (*P *= 0.001) and significant differences in community structure between treatments (*P *= 0.024).

**Figure 5:**
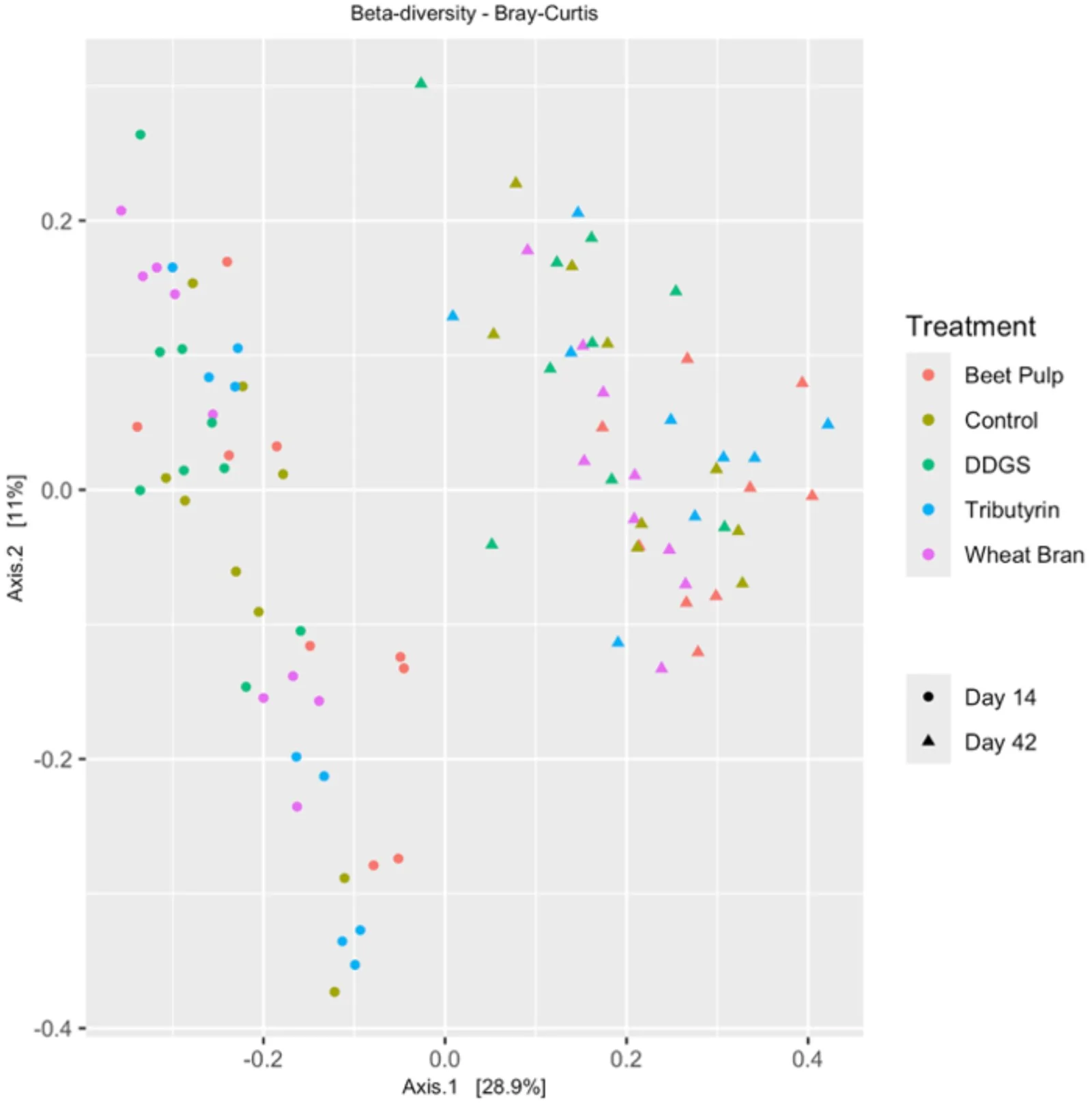
Pooled pen fecal samples were collected on d 14 and 42 post-weaning to evaluate differences in microbial community composition among dietary treatments (Control, Beet Pulp, DDGS, Tributyrin, and Wheat Bran) and between time points (D 14 and D 42). Beta diversity was assessed using Bray-Curtis dissimilarity and PERMANOVA, and principal coordinates analysis (PCoA) was used to visualize these differences. Each point represents an individual sample and is colored by dietary treatment: Shaped by time point equates to d 14 (circle) and d 42 (triangle); Control (yellow), Beet Pulp (orange), DDGS (green), Wheat Bran (pink), and Tributyrin (blue). Axes represent the principal coordinates derived from Bray-Curtis distance values, capturing variation in microbial composition among samples. Distinct clustering was observed between d 14 and 42 (*P *= 0.001), and PERMANOVA identified significant differences in bacterial community composition among dietary treatments (*P *= 0.024).

Given the fecal bacterial community composition differed between dietary fiber treatments, further analysis of differentially abundant ASVs between different fiber sources was performed (Figure 6) using DESeq2 package in R. Differential analysis between BP and DDGS identified several ASVs that were significantly different, including ASVs belonging to *Lactobacillus*, *Provotellaceae*, and *Rikenellaceae* enriched in BP, and *Muribaculaceae* enriched in DDGS (*P *< 0.05).

**Figure 6:**
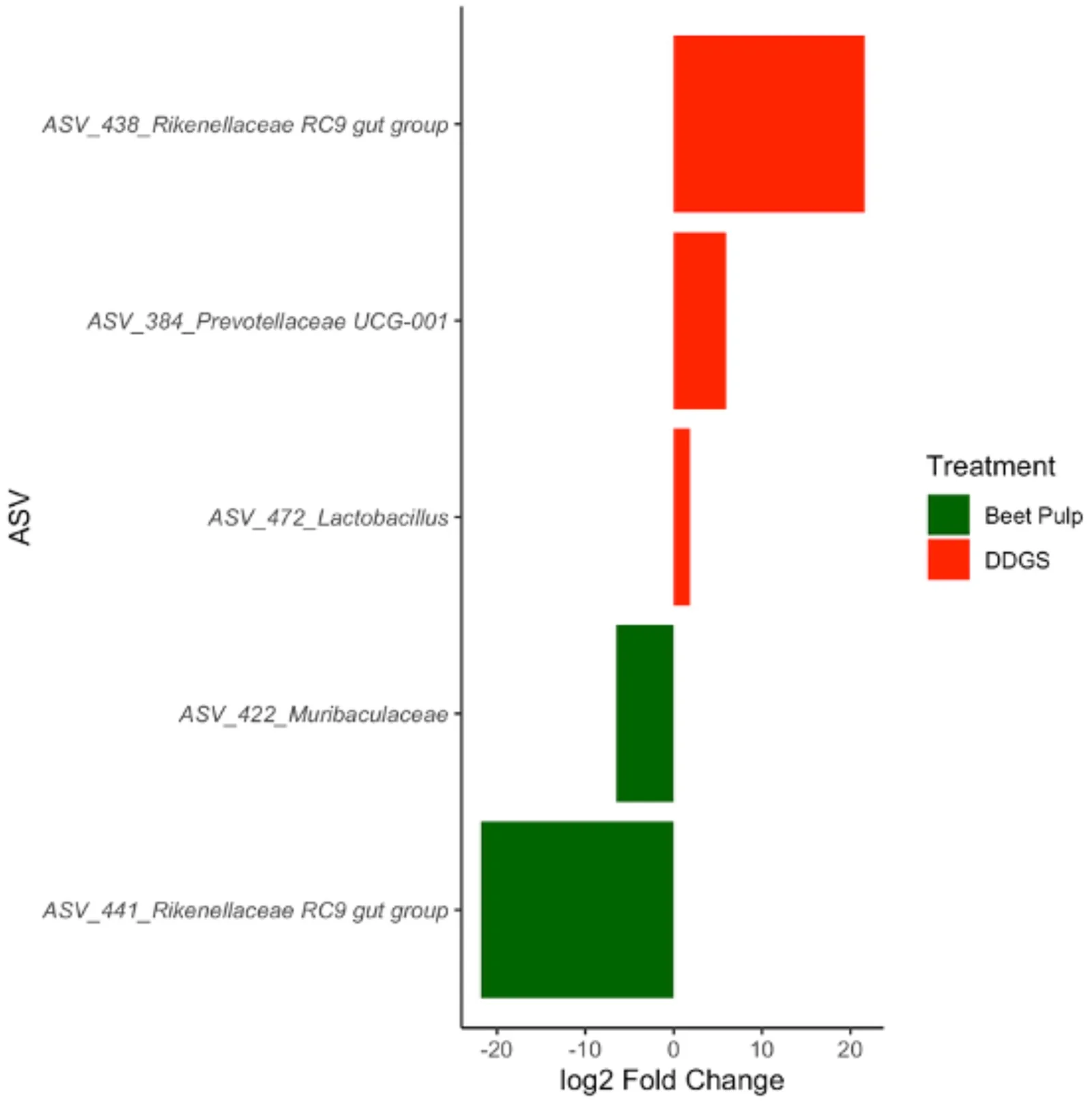
Pooled pen fecal samples were collected on d 42 to evaluate differential abundance of bacterial genera and associated ASVs between Beet Pulp and DDGS treatments. Each bar represents a bacterial genus and its associated ASV, colored by dietary treatment: Beet Pulp (green) and DDGS (red). Bar length reflects the magnitude of difference between treatments. Bacterial genera and associated ASVs with positive linear discriminant analysis (LDA; log_2_) scores are enriched in DDGS, whereas those with negative LDA scores are enriched in Beet Pulp.

## Discussion

The response of nursery pigs to dietary fiber is multifactorial, where insoluble fiber sources primarily modulate gut function and health through physical and kinetic effects in the small intestine, and soluble fiber sources influence hindgut fermentation and VFA production (Jha and Berrocoso 2015). In the present study, we evaluated whether dietary inclusion of fiber sources (WB, sugar BP, and corn DDGS) and tributyrin (a direct butyrate source) could improve intestinal health, microbial composition, VFA concentration, and growth performance in nursery pigs. Corn DDGS contains 36.1% TDF, comprising 33.7% insoluble and 2.4% soluble fiber. WB contains a similar TDF content of 39%, with 36.5% insoluble and 2.5% soluble fiber. In contrast, sugar BP has a higher TDF content of 64.0%, a substantially greater soluble fiber fraction of 14.9%, and an insoluble fiber fraction of 49.1% (Acosta et al. 2026). Acosta et al. (2026) also reported that the insoluble to soluble dietary fiber ratio for wheat bran, corn DDGS and BP is 14.6, 14.0, and 3.3, respectively. Tributyrin was included as a complementary nutritional strategy to directly deliver butyrate to the small intestine following enzymatic hydrolysis (Claus et al. 2007).

A critical context of this study was the presence of a naturally occurring mixed enteric pathogen burden, consisting of rotavirus and hemolytic F18 ETEC. This multi-pathogen burden is common in the early nursery period in U.S. commercial pig production (Cezar et al. 2026). The poor nursery performance reported herein across all dietary treatments is likely explained by this poor enteric health, as these weaned pigs are most vulnerable to low feed intakes, epithelial damage, and dysbiosis (de Mille et al. 2022). Independent of enteric pathogen burden, high fiber diets have been shown to reduce growth rates in nursery pigs, as these diets are bulkier and increase gut fill (Ndou et al. 2013; Agyekum and Nyachoti 2017). Avelar et al. (2010) reported reductions in ADFI, ADG, and feed efficiency when increasing wheat DDGS inclusion from 0% to 20% in nursery pigs despite diets being formulated to similar net energy and standard ileal digestibility amino acids. However, in the current study, the fiber-containing diets (WB, BP, and DDGS) had a lower inclusion rate and no ADG, ADFI, and G:F were reported compared to the CON. This finding is consistent with Batson et al. (2021), who reported that coarse wheat bran (4%), oat hulls (1.85%), and cellulose (1.55%) had no impact on nursery pig growth performance. Weber and Kerr (2008), reported that supplementation up to 0.4% sodium butyrate had no effect on final BW or feed efficiency, but decreased ADFI in pigs. Interestingly, compared to the CON, TB tended to increase ADFI during phase 2 and overall, without affecting ADG or BW. Similarly, Gálfi and Bokori (1990) reported that supplementing pigs with 0.17% sodium *n*-butyrate increased ADFI and ADG, and improved ileal and cecal morphology, suggesting enhanced surface area for nutrient absorption. Mendoza (2011) reported increased villus height in the jejunum with corn DDGS supplementation; however, no differences in intestinal morphology were observed across treatments in the current study.

Molist et al. (2010) demonstrated that the physical form and particle size of wheat bran, rather than simply its inclusion level, played a critical role in modulating enteric health outcomes in nursery pigs. In particular, their work showed that coarse wheat bran reduced K88 ETEC adhesion in the ileum, whereas finely ground wheat bran did not produce the same effect (Molist et al. 2010). Further, Li et al. (2019) demonstrated reduced ileal attachment of *E. coli* in nursery pigs challenged with F18 ETEC and fed diets containing sugar BP, although dietary inulin and BP failed to reduce *E. coli* shedding in other challenged pigs (Halas et al. 2009; Yan et al. 2017). In the current study, nursery pigs were naturally enterically burdened with rotavirus and hemolytic ETEC. Although *F18* and *LT* gene abundance increased in d 14 fecal samples, corresponding with the period when pigs tested positive for hemolytic *E. coli*, no significant differences were detected among CON, WB, DDGS, and BP treatments in fecal abundance of either *F18* or *LT* gene abundance. Due et al. (2025) utilized this PCR method to confirm F18 ETEC in fecal samples following a control F18 ETEC challenge, reporting that both *F18* and *LT* gene abundance were elevated in F18 ETEC-challenged pigs compared to non-challenged pigs.

Although F18 and LT gene abundance measured by PCR did not differ among dietary treatments, fecal *E*. *coli* abundance determined by 16S rRNA sequencing differed, with CON and BP-fed pigs exhibiting lower relative *E*. *coli* abundance than wheat bran-fed pigs, while DDGS and tributyrin were intermediate. These findings are not necessarily contradictory because quantitative PCR and 16S rRNA gene sequencing quantify different aspects of the bacterial population. Quantitative PCR is a highly sensitive and specific method that targets virulence genes (eg F18 fimbriae and LT enterotoxin) to estimate the abundance of pathogenic F18 ETEC (Due et al. 2025), whereas 16S rRNA gene sequencing measures the relative abundance of the entire *Escherichia* genus and generally cannot distinguish pathogenic from commensal *E*. *coli* strains due to limited taxonomic resolution (Jovel et al. 2016; Pollock et al. 2018). Consequently, changes in the overall abundance of *Escherichia* spp. do not necessarily reflect changes in the burden of pathogenic F18 ETEC, which likely explains why differences in relative *E*. *coli* abundance were not accompanied by differences in F18 or LT gene abundance among treatments.

Regardless of dietary treatment, fecal consistency improved progressively throughout the nursery period, reflecting natural recovery from the post-weaning enteric challenge. Although DDGS tended to increase the proportion of loose feces and tributyrin tended to increase the proportion of solid feces during the later stages of the study, these modest differences were not accompanied by reductions in fecal F18 or LT gene abundance or improvements in growth performance, suggesting limited biological significance under the conditions of this natural rotavirus and F18 ETEC challenge. Altogether, these data suggest that there was minimal benefit of BP, WB, DDGS, and TB at the inclusion levels fed on mitigating F18 ETEC and PWD. Collectively, these inconsistencies highlight that fiber-mediated pathogen modulation is highly context-dependent, influenced by infection pressure, dietary inclusion level, timing relative to weaning, and microbial baseline stability. It is also likely that rotavirus, rather than F18 ETEC alone, contributed substantially to the observed fecal consistency changes, as this study did not include a viral load assay to differentiate the relative contributions of each pathogen to enteric disturbances. Further, rotavirus infection is expected to produce a malabsorptive diarrhea that will result in additional undigested substrates reaching the colon that are thereby available for fermentation and may also lead to changes in the microbiota.

Dietary fiber can be fermented by the hindgut microbiota to produce VFAs, which provide energy to the host and contribute to intestinal health and immune function (Asp 1996; Koh et al. 2016). Given that insoluble fiber is generally less fermentable than soluble fiber, it is often associated with lower VFA production (Jha and Berrocoso 2015). Consistent with this, Zhang et al. (2023) reported greater fecal butyrate and valerate concentrations in pigs fed sugar BP compared with corn DDGS. However, soybean hulls, despite being predominantly insoluble, also increased acetate and total VFA concentrations due to their highly fermentable oligosaccharide content (Molist et al. 2014; Zhang et al. 2023). Similarly, Molist et al. (2011) observed increased fecal butyrate, propionate, and total VFA concentrations in nursery pigs fed wheat bran. In contrast, the present study found lower fecal acetate and total VFA concentrations in WB-fed pigs than CON on d 22, with no treatment differences in ileal or colonic VFA concentrations.

These findings should be interpreted cautiously because luminal and fecal VFA concentrations represent only a small fraction of total microbial VFA production, as most VFAs are rapidly absorbed by the intestinal epithelium (Montoya et al. 2016). Furthermore, increased fecal bulk associated with insoluble fiber may dilute VFA concentrations when expressed per gram of feces (Tungland and Meyer 2002). Consistent with our findings, Chen et al. (2013) also reported no differences in ileal VFA concentrations in pigs fed wheat bran despite observing greater colonic butyrate production. The observed increase in VFAs over time across all treatments likely reflects normal maturation of microbial fermentative capacity following weaning, rather than a direct dietary effect. This temporal pattern is consistent with prior work showing progressive stabilization of microbial fermentation as pigs adapt to solid feed and gut microbiota communities mature (Luo et al. 2022). However, the absence of consistent VFA responses across dietary treatments indicates that differences in fermentability did not translate into substantial shifts in metabolic output.

Tributyrin was included in the diet to bypass limitations associated with microbial fermentation by delivering the VFA butyrate directly to the small intestine, where ETEC colonization and intestinal dysfunction primarily occurs in nursery pigs (Due et al. 2025). Butyrate has well-documented effects on epithelial proliferation, tight junction regulation, and anti-inflammatory signaling pathways (Hamer et al. 2008; Guilloteau et al. 2010). However, in the present study, TB did not improve villus architecture, reduce pathogen burden, or enhance growth performance. One possible explanation is that TB hydrolysis and butyrate release may not have occurred at sufficient concentration or in the appropriate intestinal segments to exert functional effects. Alternatively, the severity of epithelial disruption induced by weaning transition and pathogen burden may have exceeded the capacity of butyrate-mediated repair mechanisms. It is also possible that the benefits of butyrate would be more evident under subclinical or preventive conditions rather than during active acute disease (Weber and Kerr 2008).

It was hypothesized that inclusion of both insoluble and soluble fiber sources would shift the bacterial community toward beneficial taxa such as *Lactobacillus*, which supports enteric health and improved growth performance in nursery pigs. A key finding of this study was that dietary fiber influenced microbial community composition (beta diversity) without altering alpha diversity metrics. In other words, dietary fiber did not create new microbial diversity but selectively modulated existing ecological niches. This pattern supports the concept that dietary interventions in pigs more commonly shifts relative abundance of taxa rather than overall richness or evenness (Wang et al. 2025). The increase in *Lactobacillus* abundance at d 14, followed by a decline at d 42, reflects expected microbial succession following weaning. Early post-weaning conditions favor lactic acid bacteria due to simple carbohydrate availability and reduced microbial stability, whereas later stages promote expansion of more complex carbohydrate degraders such as *Streptococcus* and *Segatella* (Ke et al. 2019; Han et al. 2024).

Dietary effects were evident at finer taxonomic resolution, with BP increasing fiber-degrading taxa such as *Prevotellaceae* and *Rikenellaceae*. These organisms are strongly associated with pectin and hemicellulose degradation and VFA production, particularly acetate and propionate (Patil et al. 2020; Niu et al. 2022). In contrast, DDGS enriched *Muribaculaceae*, a group increasingly recognized for its capacity to degrade complex polysaccharides and participate in fiber utilization in monogastric systems (Zhu et al. 2024).

One of the central observations of this study was the disconnect between microbial compositional shifts and host-level outcomes. Despite these compositional changes, functional host phenotypic outputs (growth, morphology, VFA) were not consistently altered, suggesting a degree of functional redundancy within the microbial ecosystem. This supports the emerging concept that microbial community structure can shift substantially without proportional changes in metabolic output, particularly in systems under environmental or infectious stress (Liu et al. 2025). Further, this highlights that microbial changes alone are insufficient to infer functional outcomes without concurrent improvements in host physiology. The nursery phase may also represent a biologically constrained window for eliciting microbial community benefits from dietary fiber supplementation. During the immediate post-weaning period, feed intake is low, digestive and enzymatic capacity is still developing, and the intestinal microbial ecosystem is highly dynamic. These factors may limit the extent to which dietary fiber is fermented or microbial metabolites accumulate to concentrations sufficient to influence host physiology and pathogen loads, potentially explaining the lack of treatment effects observed for several parameters in the present study. In contrast, greater responses to dietary fiber are often observed in grow-finish pigs or sows, where feed intake is greater, microbial communities are more established, and fermentation capacity is more developed (Luo et al. 2022). Thus, introducing fiber-based interventions later in the nursery period or near the transition to grow-finish may provide a more favorable biological environment for eliciting measurable microbial and host responses. However, this potential advantage must be considered in the context of the immediate post-weaning period, which is also characterized by substantial dietary, environmental, and microbial challenges and increased susceptibility to enteric pathogens. Therefore, the limited response to fiber observed in the present study should not necessarily be interpreted as evidence that fiber is ineffective during the immediate post-weaning period, but rather that this window may require fiber sources, inclusion levels, or combinations with other dietary strategies specifically designed to overcome the constraints associated with low feed intake and an immature and rapidly changing gastrointestinal ecosystem. Future research should determine whether targeted fiber interventions introduced immediately after weaning can provide meaningful pathogen resilience, while later supplementation may be better suited for maximizing fermentation-derived microbial and host performance benefits.

Overall, the data indicate that dietary fiber source and tributyrin supplementation primarily modulated microbial community structure without substantially altering host performance or intestinal health under conditions of natural rotavirus and ETEC burden. Based on the findings of this study, it can be concluded that the inclusion of wheat bran, BP, corn DDGS, or tributyrin did not exert significant effects on intestinal morphology, F18 *E. coli* abundance, VFA concentration, or overall growth performance in nursery pigs. These results contradict the initial hypothesis that both insoluble and soluble dietary fiber sources, along with tributyrin, would enhance performance and enteric health in nursery pigs.

## Data Availability

Data available upon reasonable request to the corresponding author.

## Abbreviations

ADG: average daily gain
ADFI: average daily feed intake
ASV: amplicon sequence variants
BP: Beet pulp
BW: body weight
CON: Control diet
DDGS: corn distiller’s dried grains with solubles
ETEC: enterotoxigenic Escherichia coli
GC-MS: gas chromatography–mass spectrometry
G:F: gain:feed
NDF: neutral detergent fiber
PWD: post-weaning diarrhea
TB: tributyrin
VFA: volatile fatty acid
WB: wheat bran
